# Singapore’s Dengue Outbreak Amidst the COVID-19 Pandemic: Challenges, Responses, and Lessons

**DOI:** 10.2147/IDR.S397407

**Published:** 2023-02-22

**Authors:** Huzaifa Ahmad Cheema, Rustam Shariq Mujtaba, Amna Siddiqui, Laiba Imran Vohra, Abia Shahid, Jaffer Shah, Abdulqadir J Nashwan, Natasha Howard

**Affiliations:** 1 Division of Infectious Diseases, Department of Medicine, King Edward Medical University, Lahore, Pakistan; 2 Department of Pharmacy, National University of Singapore, Singapore, Singapore; 3 Department of Medicine, Karachi Medical and Dental College, Karachi, Pakistan; 4 Department of Medicine, Ziauddin University, Karachi, Pakistan; 5 New York State Department of Health, Albany, NY, USA; 6 Hamad Medical Corporation, Doha, Qatar; 7 Saw Swee Hock School of Public Health, National University of Singapore and National University Health System, Singapore, Singapore; 8 Free Aleppo University, Aleppo, Syria; 9 Department of Global Health and Development, London School of Hygiene & Tropical Medicine, London, UK

**Keywords:** DENV-3, SARS-CoV-2, infectious disease control, Singapore

## Abstract

Dengue outbreaks have been documented in Singapore since 1901, occurring almost annually in the 1960s and disproportionately affecting the paediatric population. In January 2020, virological surveillance detected a shift from DENV-2, which was the previous dominant strain, to DENV-3. As of 20 September 2022, 27,283 cases have been reported in 2022. Singapore is currently also responding to the COVID-19 pandemic, overcoming another wave of infections with 281,977 cases recorded in the past two months as of 19 September 2022. While Singapore has adopted several policies and interventions to combat dengue, primarily through environmental control but also innovations such as the Wolbachia mosquito programme, there is a need for further efforts to deal with the dual threat of dengue and COVID-19. Drawing lessons from Singapore’s experience, countries facing such dual epidemics should enact clear policy responses, including establishing a multisectoral dengue action committee and action plan prior to potential outbreaks. Key indicators should be agreed upon and tracked at all healthcare levels as part of dengue surveillance and incorporated into the national health information system. Digitizing dengue monitoring systems and implementing telemedicine solutions are innovative measures that would facilitate the response to dengue in the context of restrictions during the COVID-19 pandemic that hinder the detection and response to new cases. There is a need for greater international collaboration in reducing or eradicating dengue in endemic countries. Further research is also required on how best to establish integrated early warning systems and extend our knowledge of the effects of COVID-19 on dengue transmission in affected countries.

## Commentary

Dengue is caused by the dengue virus, existing as four different serotypes of single-stranded, positive-sense RNA viruses (DENV-1 to DENV-4), causing over 96 million symptomatic infections per year.^1^ A dengue infection confers lifelong immunity only to that serotype and temporary cross-immunity against other serotypes.^1^ However, when cross-immunity wanes, a secondary infection can lead to life-threatening conditions such as dengue haemorrhagic syndrome and dengue shock syndrome.^1^

Dengue outbreaks have been documented in Singapore since 1901, occurring almost annually in the 1960s and disproportionately affecting the paediatric population.^2^ In response, the Singapore government targeted *Aedes* mosquitoes, the primary vector of dengue, and successfully reduced the incidence of dengue by about 90%.^3^ However, this successful vector control contributed to reduced levels of population immunity, leading to new outbreaks.^3^ With persistently high case numbers, dengue has remained hyperendemic in Singapore for the past 30 years.^3^

Data suggest Singapore experiences dengue epidemics in multi-annual cycles, with larger surges attributed to a switch in the predominant serotype.^2^ This cyclical pattern was found to be crucial in the 2004–2007 and 2013–2016 waves of infections, where the epidemic peak was preceded by a change from DENV-2 to DENV-1.^2^ In January 2020, virological surveillance detected a shift from DENV-2 to DENV-3.^4^ As DENV-3 was not predominant in Singapore for almost thirty years, population immunity was low. Despite pre-emptive measures 2020 documented over 35,000 cases, the highest in Singapore’s history.^4^ In 2022, DENV-3 remains the dominant serotype with 27,283 cases being reported as of 20 September 2022.^4^ With the dengue season stretching from June to October, the Singapore government is working to counter rising case numbers.

Dengue control is spearheaded by National Environment Agency (NEA), collaborating with private, governmental, and academic stakeholders in implementing an integrated vector-control programme.^5^ This approach relies heavily on source reduction and larviciding, such as by conducting regular inspections in construction sites and using *Bacillus thuringiensis* subsp. *israelensis* (BTi) and *Wolbachia* bacteria in mosquito breeding hotspots.^5^ NEA also works closely with the Ministry of Health (MOH) and research institutions to conduct laboratory-based dengue virus surveillance to track circulating dengue serotypes among cases, utilising such data to develop dengue risk prediction models.^5^,^6^

Singapore is currently also responding to the COVID-19 pandemic, overcoming another wave of infections with 281,977 cases recorded in the past two months as of 19 September 2022.^7^ A recent study demonstrated a strong positive correlation between COVID-19 and dengue cases in Asian countries, underscoring the possible impact of the COVID-19 pandemic on the burden of dengue.^8^ This indicates the need for greater vigilance to counter the growing dengue threat. Our commentary focuses on Singapore and highlights the lessons that can be learnt from the ongoing dengue outbreak.

## Challenges and Mitigation Efforts

With dengue endemic, Singapore has adopted long-term policies and interventions to combat dengue, primarily through environmental control but also innovations such as the Wolbachia mosquito programme, as summarised in Table 1. Wolbachia-infected mosquitoes have proved to be effective in reducing the incidence of dengue as well as decreasing the risk of hospitalization due to dengue, and their application as a public health intervention in dengue-endemic areas appears to be suitable.^9^ In Singapore, preliminary evidence shows that this strategy is highly successful in controlling the spread of dengue by suppressing the *Aedes aegypti* population and decreasing the number of dengue cases.^10^

**Table 1:** Summary of Dengue Control Efforts Adopted by the Singaporean Government

Initiative	Aim
Mosquito control	
National Dengue Prevention Campaign^5^	Launched in March by the National Environment Agency (NEA), ahead of the usual May launch coinciding with the start of “dengue season”, to mobilise the nation to be conscientious in housekeeping, remove stagnant water, and deprive *Aedes* mosquitoes of breeding habitats.
NEA Wolbachia Mosquito factory in Ang Mo Kio, Singapore^5^	NEA produces up to two million Wolbachia mosquitoes weekly for release and aims to breed about five million weekly. Male mosquitoes infected with Wolbachia bacteria are released to mate so resultant eggs cannot produce offspring, making the wild population crash. Multi-year data from pilot sites in Tampines and Yishun suggest the approach successfully reduced mosquito populations there.
Dengue vaccine	
Dengvaxia^23^	Singapore’s Health Sciences Authority states this vaccine is approved for use only for Singaporeans aged 12–45 who have already been infected with at least 1 dengue strain and requires three doses administered over 12 months.
Legislative measures	
Infectious Diseases Act (IDA); Control of Vectors and Pesticides Act (CVPA); Environmental Public Health Act (EPHA)	These acts prescribe significant penalties for anyone failing to comply with the law. IDA deals with notification, investigation and treatment of dengue; CVPA deals with vector control; and EPHA deals with environmental sanitation and other environmental public health issues.

Furthermore, Singapore has developed a comprehensive response framework that includes legislating dengue environmental control through the Infectious Disease Act (IDA), Control of Vectors and Pesticides Act (CVPA) and the Environmental Public Health Act (EPHA).^11^ However, further preventative measures may be needed as a suitable vaccine is not yet ready for market. Dengvaxia is available in Singapore but concerns about potential risks and eligibility constraints have prevented its large-scale adoption as Singaporeans wait for a better alternative.^12^ Echoing this sentiment, the Singapore Ministry of Health’s Expert Committee on Immunisation advised in 2020 that Dengvaxia was not effective to control dengue at the population level and would not be considered for national immunisation schedule subsidy.^13^ Other vaccines are also under development including promising candidates by Takeda and the US’s National Institute of Health. The Takeda vaccine is being considered for approval by the European Medicines Authority and it will be essential for Singaporeans to assess its suitability here given the state’s unique epidemiology.^12^ Vaccines remain an integral part of public health intervention and with the demonstrated effectiveness of Singapore’s national COVID-19 vaccination roll-out, optimism is warranted that dengue vaccination could be widely implemented once a suitable vaccine becomes available.

Research indicates that temperature can influence the transmissibility of dengue as warmer conditions are associated with faster development and increased survivability of *Aedes* mosquitoes as well as faster viral replication within infected mosquitoes.^14^ Due to climate change, increasing temperatures are predicted to make Singapore more vulnerable to mosquitoes year-round.^15^ This introduces additional challenges to the health system already burdened by COVID-19. Furthermore, low dengue infection over the past years has meant population immunity in Singapore is insufficient. The present epidemic appears predominantly due to DENV-3, a less researched dengue strain with a low reported incidence in Singapore for almost three decades, resulting in extremely low herd immunity.^4^

Differentiating between COVID-19 and dengue infection is challenging without diagnostic testing, as many initial clinical symptoms overlap. Both present as a fever associated with nonspecific signs and symptoms. Thus, such patients are triaged as potential COVID-19 until proven otherwise via diagnostic testing.^16^,^17^ Polymerase chain reaction (PCR) testing for SARS-COV-2 is the most reliable but takes 24 hours or more, thus delaying diagnosis. Rapid diagnostic tests (RDTs), such as serological tests and rapid antigen detection tests, are quicker for dengue and SARS-COV-2 diagnosis respectively, enabling rapid response management for patients. However, RDTs can yield false-negative and false-positive results ensuing inadvertent exposure of health workers and other patients.^17^

## Lessons

As the world continues to concentrate on controlling the COVID-19 pandemic, persistent measures are still required to mitigate endemic infections and minimise the emergence or re-emergence of other pathogens. The COVID-19 pandemic has coincided with a resurgence of dengue in countries such as Brazil, India, Malaysia, and Pakistan. Drawing lessons from Singapore’s experience, countries facing such dual epidemics should enact clear policy responses, including establishing a multisectoral dengue action committee and action plan prior to potential outbreaks. Key indicators should be agreed upon and tracked at all healthcare levels as part of dengue surveillance and incorporated into the national health information system. Due to potential changes in the dominant serotype, as seen in Singapore’s current dengue epidemic, countries should include the identification of all serotypes. Additionally, affected countries should aim to increase funds for infectious disease control. While COVID-19 mitigation topped most 2020–2022 budgets, funding for other endemic or re-emerging diseases should also increase. A dedicated budget for dengue response, accessible at peripheral and local levels as relevant, can help ensure timely action. Strengthening dengue surveillance, ensuring funding for prevention and response, conducting social mobilisation and vector control, improving diagnostic testing capacities, and health staff training on dengue and COVID-19 in the context of co-epidemics are crucial.^18^

Singapore has relied extensively on individual and community-level environmental management, suggesting social mobilisation campaigns and legislation encouraging individual action and responsibility have a role in reducing breeding sites and increasing knowledge of disease symptoms thus encouraging early health facility attendance and diagnosis. By participating in the Mozzie Wipeout, citizens may help avoid mosquito breeding in their residences, which is an important step in reducing the mosquito count. Homeowners are urged to maintain excellent housekeeping, implement vector control strategies, and take actions including routinely inspecting their dwellings for standing water and discarding any equipment that gathers water.^19^

Public involvement tactics may need to be revised in light of the present COVID-19 scenario and the fallout from the dengue outbreak. In addition to the customary home inspections and dengue leaflets, interacting with stakeholders over social media networks may become the new standard such as nationwide live Facebook updates.^20^

For health systems such as Singapore, which must respond to concurrent epidemics of dengue and COVID-19, following a strict triage algorithm while differentiating the two diseases is essential for infection prevention and control and to avoid coinfection.^16^ Owing to the similarity in the presentation of both diseases, frontline clinicians should consider co-infection and false-positive dengue serology.

Implementing telemedicine solutions, particularly in areas with less access to diagnostic facilities or care could aid access and identification of areas requiring additional epidemiological surveillance. Moreover. digitizing dengue monitoring systems should be a top priority for the governments of affected countries. Two cities in the Philippines and one in Indonesia have established mobile-based dengue monitoring systems, exhibiting encouraging findings. For every verified dengue case, these technologies will enable healthcare professionals to promptly alert local and national healthcare authorities.^21^

Finally, Singapore’s response is comprehensive and carefully planned with extensive use of data. This can be challenging for larger poorer countries to emulate. For example, a Wolbachia mosquito release strategy, while potentially effective, may be too expensive for some countries on their own. This suggests a role for international partners in providing technical and financial support.^5^ This points to the need for greater international collaboration in reducing or eradicating dengue in endemic countries. Ongoing collaborations, such as UNITEDengue,^22^ are a step in the right direction. Further research is also needed on how best to establish integrated early warning systems and extend our knowledge of the effects of COVID-19 on dengue transmission in affected countries.
